# Epidemiological study of Newcastle disease in chicken farms in China, 2019–2022

**DOI:** 10.3389/fvets.2024.1410878

**Published:** 2024-10-30

**Authors:** Shida Wang, Lili Wei, Jingfei Wang, Zhigang Zhang

**Affiliations:** ^1^College of Veterinary Medicine, Northeast Agricultural University, Harbin, China; ^2^State Key Laboratory for Animal Disease Control and Prevention and National Data Center of Animal Infectious Diseases, Harbin Veterinary Research Institute, Chinese Academy of Agricultural Sciences, Harbin, China

**Keywords:** Newcastle disease virus, prevalence, risk factor, phylogenetic analysis, random forest, China

## Abstract

Newcastle disease virus (NDV) is a significant poultry pathogen, causing ongoing economic losses in China’s poultry industry. To understand its circulation and risk factors associated with farm infection, we carried out an epidemiological study on 1,065 farms across 18 provinces from 2019 and 2022. We collected a total of 43,696 swabs and tested them for NDV using an RT-PCR that targets the partial F gene. The overall bird-level NDV prevalence in the 4 years (2019, 2020, 2021, and 2022) were 1.49% (95%CI: 1.27, 1.71%), 1.24% (95%CI: 1.04, 1.44%), 0.59% (95%CI: 0.45, 0.74%), and 0.44% (95%CI: 0.31, 0.58%) respectively, while the farm-level prevalence of the 4 years were 11.27% (95%CI: 7.93, 14.60%), 13.85% (95%CI: 10.10, 17.61%), 12.63% (95%CI: 8.00, 17.26%), and 10.71% (95%CI: 6.38, 15.04%), respectively. The result also showed a high degree of variation in farm-level prevalence (ranging from 0 to 90%) across the provinces. A multiple logistic regression analysis revealed that four factors, namely layer farm (OR = 2.282, 95% CI: 1.211, 4.298), broiler farm (OR = 2.339, 95% CI: 1.206, 4.534), using mixed feed (OR = 2.973, 95% CI: 1.962, 4.505), and indoor housing with some free-range access (OR = 1.788, 95%CI: 1.096, 2.917), increased the risk of NDV infection on farms. We isolated 104 NDVs, which were all classified into Class II by the phylogenetic analysis, but from two genotypes: four belonged to genotype I, while the remaining 100 mainly belonged to genotype II, indicating that the circulating NDVs were primarily LaSota-like low pathogenic viruses. We used random forest algorithm to established an occurrence risk model, The accuracy of the model was 90.81%. This large-scale investigation revealed NDV prevalence at bird, farm, and province levels over the 4 years. It also identified risk factors associated with farm NDV infections. The findings provide new insights into the epidemiology of NDV in China and offer references for global NDV control.

## Introduction

Newcastle disease (ND) is a highly contagious viral disease in poultry caused by Newcastle disease virus (NDV). It leads to significant economic losses due to its high morbidity and mortality rates (1). First reported in Java, Indonesia in 1926, the disease has since spread to nearly all poultry production areas worldwide (2, 3). In China, the disease has been reported in almost every province. To combat ND, attenuated Lasota-based vaccines are used on all commercial chicken farms, effectively preventing large-scale outbreaks. However, ND has not been eradicated from China’s poultry populations and remains a significant problem for the poultry industry. Therefore, consistently monitoring of its circulation is critical to the current control program.

NDV is a member of *Avulavirinae* within the *Paramyxoviridae* family (4, 5). NDV has a wide host range among avian species and can infect all domestic poultry. Infected birds are the primary sources of infection, with transmission mainly occurring through respiratory and digestive routes (6, 7). The NDV genome is a single-stranded, negative-sense, non-segmented RNA molecule that encodes seven major viral proteins, including nucleocapsid protein (NP), phosphoprotein (P), matrix protein (M), fusion protein (F), and haemagglutinin-neuraminidase (HN) (8, 9). Based on the F gene’s phylogeny, NDVs are classified into Class I and II, with Class II further divided into at least 20 genotypes (10–14). To date, all NDV genotypes have been identified in China’s poultry populations, with genotype VII being the most prevalent (11, 15).

Recently, epidemiological studies on NDV have primarily focused on the molecular identification of new isolates (11, 16), while the NDV prevalence and risk factors associated with farm infection in China have not been thoroughly examined.

Identifying risk factors is crucial for effective prevention and control of infectious diseases, including ND. Powerful tools like remote sensing (RS), Geographic Information Systems (GIS), and machine learning methods are now used to achieve this goal. Traditional methods of environmental data collection, such as manual gathering, are often challenging. Currently, many researchers use RS technology to monitor disease-related environmental factors—temperature, precipitation, and vegetation coverage—to analyze the primary spatial and temporal characteristics of affected areas (17, 18). GIS is widely used in epidemiological research to display epidemic data on maps, providing a more intuitive understanding of the spatial and temporal distribution of outbreaks. It also helps identify disease patterns and illustrate relationships between environmental factors and diseases. Machine learning methods are extensively employed to analyze data, uncover potential connections between disease occurrence and various factors, and establish risk assessment models (19).

In this study, we conducted an epidemiological study on 1,065 farms across 18 provinces of China from 2019 and 2022. The aim was to estimate the prevalence of NDV, identify the potential risk factors associated with farm NDV infections and assess the risk of disease occurrence under different farming conditions. Our findings offer an updated understanding of NDV circulation status in China, which is useful in formulating control policies.

## Materials and methods

### Study area

**Figure 1:**
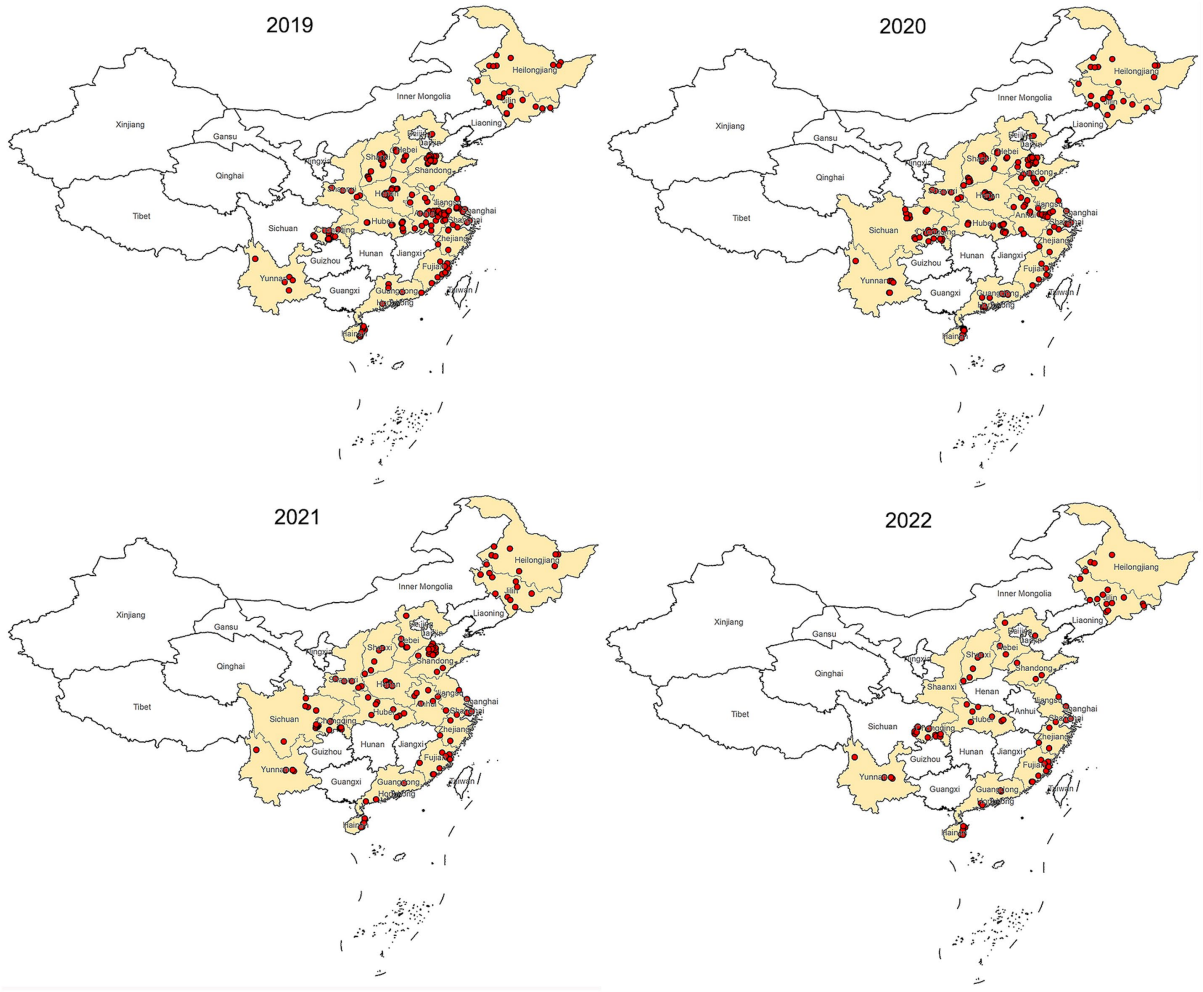
Map of China showing locations of sample collection, 2019–2022. The dots mark the farm location.

This epidemiological study was conducted from 2019 to 2022. Samples were collected from 1,065 farms across 18 provinces of China (Figure 1).

### Sample collection

**Figure 2:**
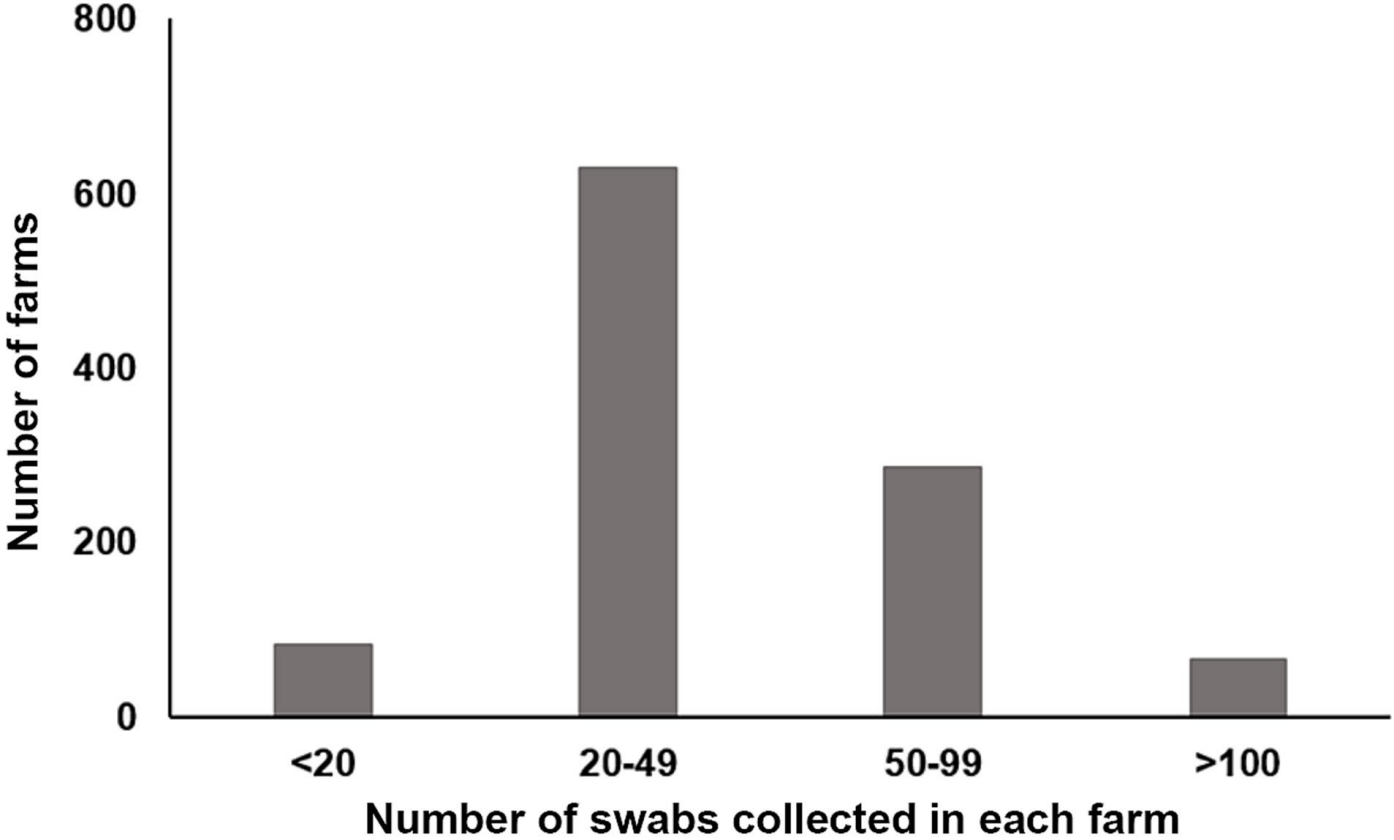
Distribution of the number of samples collected in each farm.

A total of 43,696 chicken samples, including 15,744 oropharyngeal and 27,952 cloacal swabs. The quantity of samples taken from each farm varied, ranging from eight to 200 (Figure 2). Immediately after collection, each swab was immersed in a viral medium (containing 1 mL sterile phosphate buffered saline, 2000 U/mL penicillin, 2 mg/mL streptomycin, and 10% glycerol) contained in a 2.0 mL EP tube. These samples were then frozen at −20°C for at least 2 h before being transported to our lab in a sample transport container supplied with dry ice. Detailed information about the samples is provided in Table 1.

**Table 1:** Number of farms and samples collected in the provinces from 2019 to 2022.

	2019		2020		2021		2022	
	No. of farms	No. of samples	No. of farms	No. of samples	No. of farms	No. of samples	No. of farms	No. of samples
Anhui	20	600	10	300	7	210	0	0
Fujian	12	614	10	607	16	629	14	679
Guangdong	5	500	11	600	5	600	5	600
Hainan	24	600	18	600	12	635	21	637
Hebei	24	600	18	450	9	450	6	300
Henan	32	888	20	600	10	600	0	0
Heilongjiang	16	920	20	901	15	750	10	600
Hubei	12	603	24	602	8	600	16	480
Jilin	14	600	14	600	10	600	12	600
Jiangsu	30	910	24	920	22	900	22	900
Shandong	75	1820	52	1800	25	900	30	1,200
Shanxi	32	567	30	600	10	600	10	420
Shaanxi	5	600	10	600	10	600	10	600
Shanghai	3	300	6	300	3	300	3	300
Yunnan	6	600	10	600	5	600	10	600
Zhejiang	14	599	14	600	7	630	7	482
Chongqing	22	702	24	633	19	601	20	857
Sichuan	0	0	10	300	5	600	0	0
Total	346	12,023	325	11,613	198	10,805	196	9,255

### Questionnaire

During sample collection, the investigator administered a questionnaire to collect data about the farm. The information collected mainly included the date of sampling, farm location, farm size, type of feed, feed source, water source, water disinfection methods, excrement management, and treatment of dead animals.

### RNA extraction and RT-PCR

To extract viral RNA from the samples, the swabs were vortexed for 30 s, the cotton swabs were discarded, and the samples were then centrifugated at 3,000 g for 5 min. A 200 μL volume supernatant was used for viral RNA extraction using the EasyPure Viral DNA/RNA Kit (Transgen, China) The partial F gene of NDV was detected using an RT-PCR designed to amplify a 535 bp fragment with a forward primer 5’-ATGGGCYCCAGAYCTTCTAC-3′ and a reverse primer 5’-CTGCCACTGCTAGTTGTGATAATCC-3′. The detection process used the EasyScript One-step RT-PCR SuperMix (Transgen, China) under the following conditions: transcription at 42°C for 45 min, initial PCR activation for 3 min at 95°C, denaturation at 94°C for 30 s, annealing at 55°C for 30 s, extension at 72°C for 45 s with 30 cycles, and a final extension at 72°C for 7 min. The PCR products were tested by agarose gel electrophoresis.

### Positive farm determined

A farm is considered positive for NDV if at least one sample tests positive in the RT-PCR test.

### Virus isolation and sequence analysis

To isolate NDV, 428 positive samples were incubated in specific-pathogen-free (SPF) embryonated chicken eggs. In brief, three eggs were inoculated with 0.2 mL of each sample via the allantoic route. The eggs were observed every 12 h and allantoic fluids were collected after 48 h for hemagglutination assay (HA). The HA-positive allantoic fluids were then amplified using the previously described RT-PCR method, and the PCR products were sequenced.

To analyze the phylogeny of the NDVs isolated in this study, we downloaded the F gene sequences of 34 reference NDVs from the GenBank database. We aligned all the sequences using the ClustalW algorithm implemented in MEGA 7 (20). We then built a maximum-likelihood tree with 1,000 bootstrap replicates.

### Statistical analysis

The questionnaire and PCR results were organized and summarized as numbers and percentages to estimate the prevalence of NDV by year and region. Initially, a univariate analysis was performed on the variables. Any variables with a *p*-value of less than or equal to 0.3 were included in the multivariable regression. A p-value less than 0.05 was considered statistically significant, and less than 0.01 was considered highly significant. All data analyses were conducted using SPSS (Version 19).

### Construction of risk model for occurrence

An occurrence risk model was established using random forest algorithm in the R package caret.^1^ The model randomly selected 70% data from 1,065 farms data set for training and the remaining 30% data used to validate the model. The random forest algorithm training multiple decision trees to generated a model and evaluated the model using a 10-fold cross-validation method.

## Results

### The prevalence of NDV in China from 2019 to 2022

**Figure 3:**
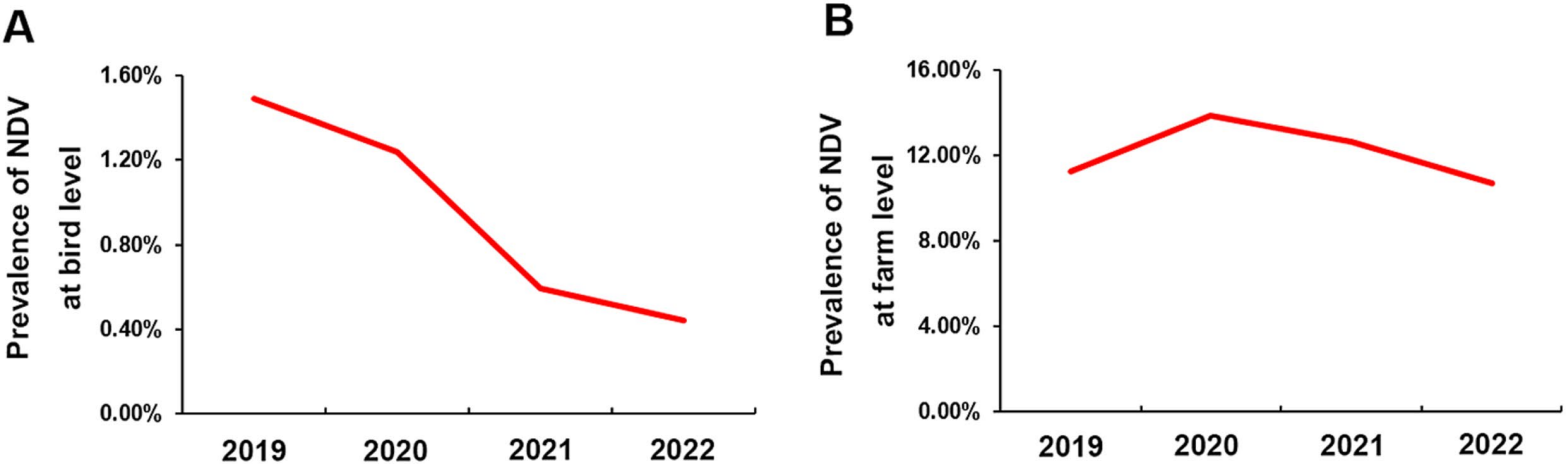
Prevalence of NDV in China from 2019 to 2022. **(A)** Bird-level NDV prevalence. **(B)** Farm-level NDV prevalence.

We used an RT-PCR method to test 43,696 swab samples for the presence of NDV, as described in the method section. We found that 0.98% (428/43,696) tested positive for the virus’s F gene. The bird-level prevalence between 2019 and 2022 were 1.49% (95%CI: 1.27, 1.71%), 1.24% (95%CI: 1.04, 1.44%), 0.59% (95%CI: 0.45, 0.74%), and 0.44% (95%CI: 0.31, 0.58%), respectively, as depicted in Figure 3A. The farm-level prevalence for the 4 years were 11.27% (95%CI: 7.93, 14.60%), 13.85% (95%CI: 10.10, 17.61%), 12.63% (95%CI: 8.00, 17.26%), and 10.71% (95%CI: 6.38, 15.04%), respectively, as shown in Figure 3B. These results suggest a gradual decrease in NDV infection in chickens over the four-year period in China. However, infection rates at the farm level remained relatively stable.

### Spatial distribution of NDV positive farms

**Figure 4:**
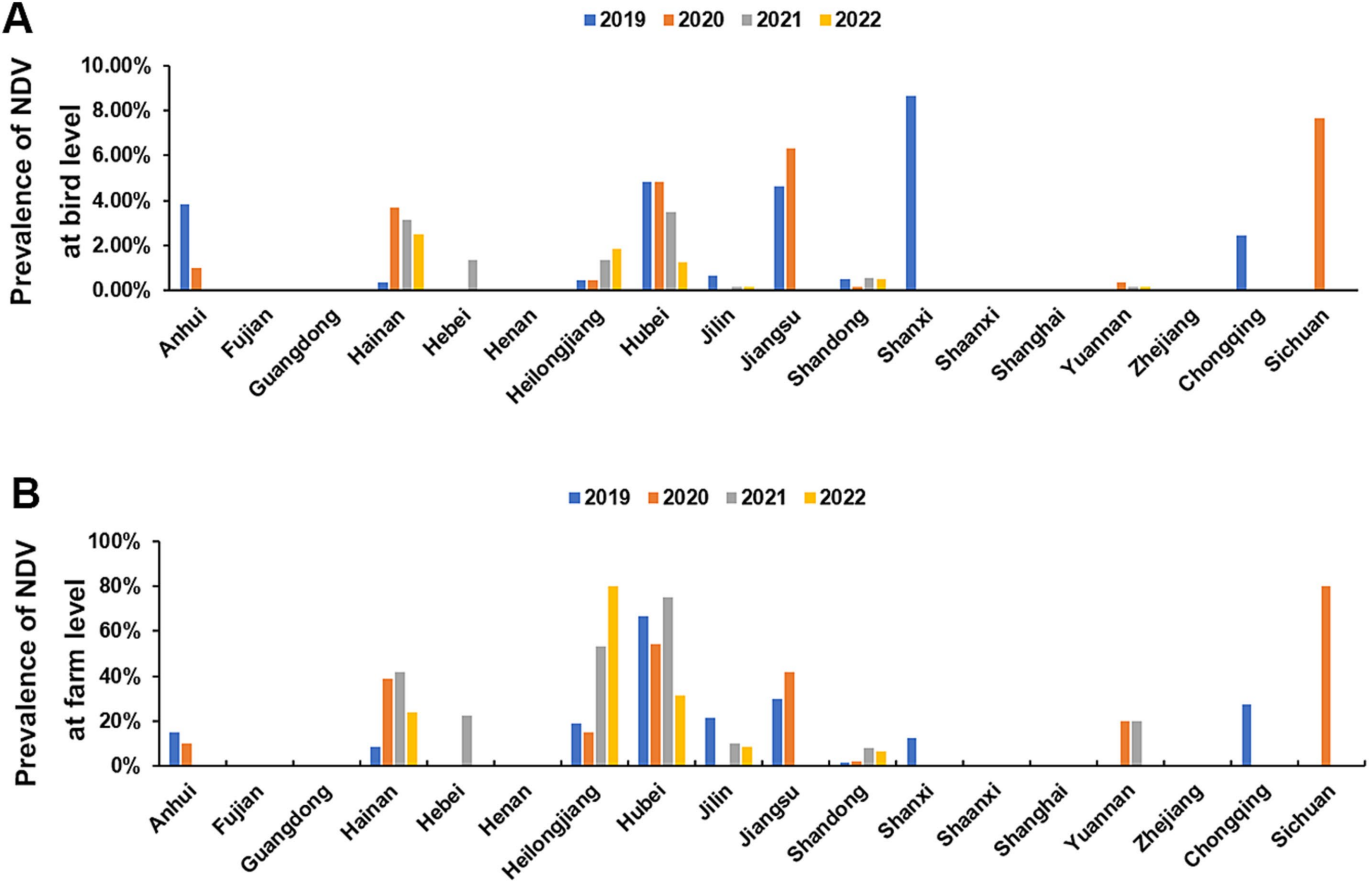
Prevalence of NDV in different provinces in China. **(A)** Bird-level NDV prevalence by provinces. **(B)** Farm-level NDV prevalence by provinces.

We found a high variation in the farm-level prevalence (ranging from 0 to 90%) among provinces (Figure 4A), yet bird-level prevalence remained relatively stable (ranging from 0 to 10%) in the provinces (Figure 4B). This suggests that low-level NDV infection is widespread across many farms in certain provinces.

**Figure 5:**
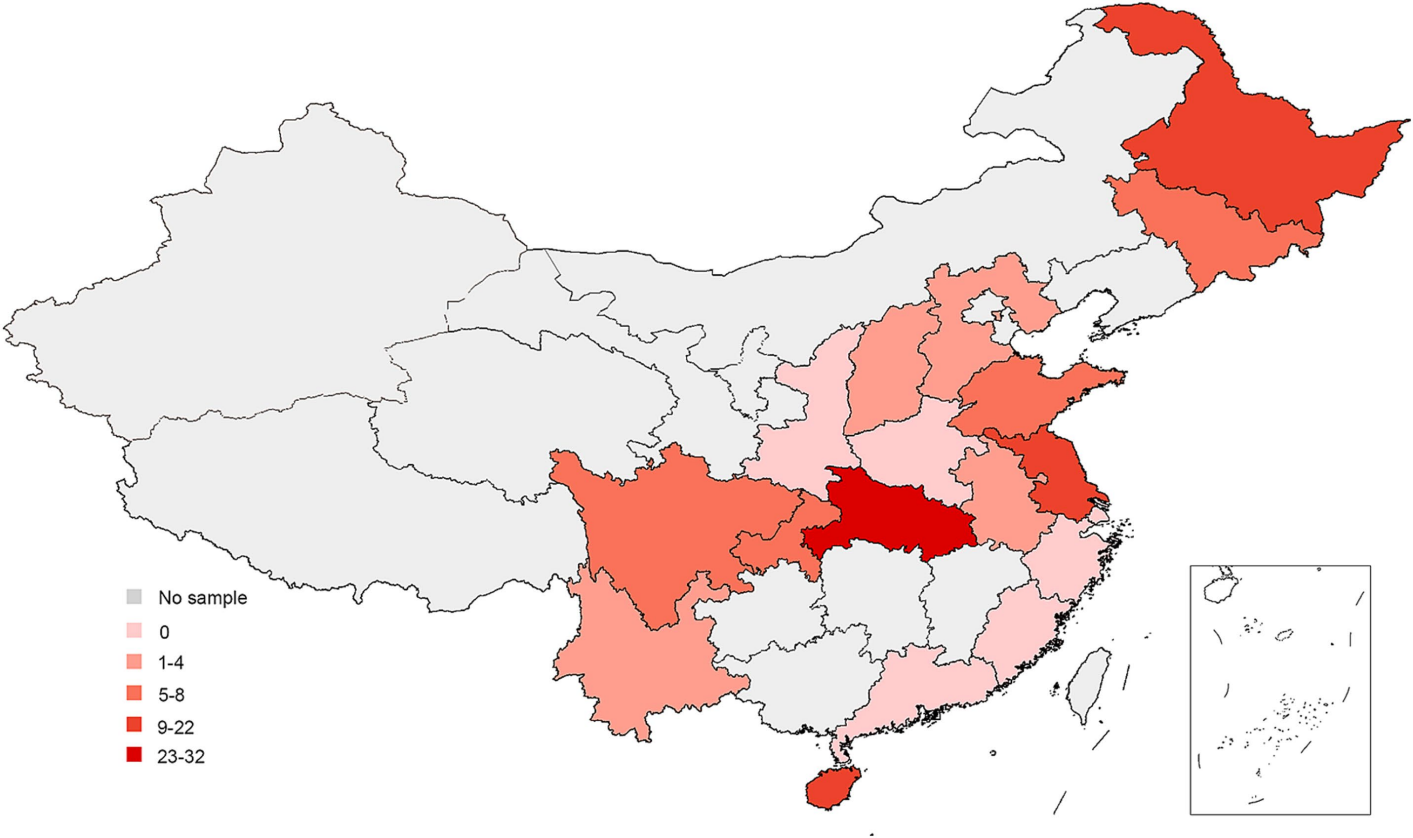
Spatial distribution of NDV Positive farms.

The most of positive farms were found in Heilongjiang, Hainan, Hubei and Jiangsu provinces. Some provinces such as Fujian, Guangdong, Henan, Shaanxi, Shanghai, Zhejiang has no NDV positive farm detected for 4 consecutive years (Table 2). This data reveals a diverse NDV circulation status across different regions in China (Figure 5).

**Table 2:** Number of positive farms and samples collected in the provinces from 2019 to 2022.

	2019		2020		2021		2022	
	No. of farms	No. of samples	No. of farms	No. of samples	No. of farms	No. of samples	No. of farms	No. of samples
Anhui	3	23	1	3	0	0	0	0
Fujian	0	0	0	0	0	0	0	0
Guangdong	0	0	0	0	0	0	0	0
Hainan	2	2	7	22	5	20	5	16
Hebei	0	0	0	0	2	6	0	0
Henan	0	0	0	0	0	0	0	0
Heilongjiang	3	4	3	4	8	10	8	11
Hubei	8	29	13	29	6	21	5	6
Jilin	3	4	0	0	1	1	1	1
Jiangsu	9	42	10	58	0	0	0	0
Shandong	1	9	1	3	2	5	2	6
Shanxi	4	49	0	0	0	0	0	0
Shaanxi	0	0	0	0	0	0	0	0
Shanghai	0	0	0	0	0	0	0	0
Yunnan	0	0	2	2	1	1	0	1
Zhejiang	0	0	0	0	0	0	0	0
Chongqing	6	17	0	0	0	0	0	0
Sichuan	0	0	8	23	0	0	0	0
Total	39	179	45	144	25	64	21	41

### Farms characteristics of NDV infection

We obtained the situation of farms by the questionnaire. Among these farms, 15.95% broiler farms were positive, 18.04% indoor housing with some free-rang access, 23.50% mixed feed, 12.74% drinking well water and 13.73% did not clean water farms were positive. We calculate the p-value of NDV infection in farms under different factors. Following the methods mentioned earlier, we ultimately selected five factors include farm type, housing, feed type, feed source and clean water for subsequent risk factor estimate. Detailed information about the farms is provided in Table 3. Association of farm level risk factors with ND infection

**Table 3:** Prevalence of Newcastle disease virus by farms characteristics.

Factors	Categories	No. of positive/tested	Prevalence (%)	*P*-value
Farm type	Layer farm	70/581	12.05	0.016
	Broiler farm	48/301	15.95	
	Breeding farm	13/183	7.10	
Housing	Indoor housing with some free-range access	35/194	18.04	<0.01
	Intensive	96/871	11.02	
Feed type	Mixed feed	51/271	23.50	<0.01
	Formula feed	80/848	9.43	
Feed source	Exotic	79/702	11.25	0.148
	Local	52/363	14.32	
Water source	Wells	116/832	12.74	0.711
	Lake water	2/19	10.53	
	Tap water	23/214	10.75	
Clean water	None	24/259	9.27	0.188
	Disinfection	81/590	13.73	
	Filter	26/216	12.04	
Waste management	Sale	46/350	13.14	0.558
	Ferment	85/715	11.89	
Dead poultry disposal	Harmless treatment	46/443	10.38	0.395
	Bury	85/622	13.67	

We conducted logistic regression analyses to identify the potential risk factors associated with NDV infection on farms. Initially, we calculated the *p*-value of all the factors and selected those with an *p* < 0.3 for the final multiple logistic regression model calculation. The model revealed an increased risk of NDV infection was associated with layer farm (OR = 2.282, 95% CI: 1.211, 4.298), broiler farm (OR = 2.339, 95% CI: 1.206, 4.534), use of mixed feed (OR = 2.973, 95% CI: 1.962, 4.505), indoor housing with some free-range access (OR = 1.788, 95%CI: 1.096, 2.917) (Table 4).

**Table 4:** Logistic regression analysis of risk factors for NDV infection.

Variable	Condition	OR	95%CI	*P*-value
Farm type	Layer farm	2.282	1.211–4.298	0.011
	Broiler Farm	2.339	1.206–4.534	0.012
	Breeding farm	Ref		
Housing	Indoor housing with some free-range access	1.788	1.096–2.917	0.020
	Intensive	Ref		
Feed type	Mixed feed	2.973	1.962–4.505	<0.01
	Formula feed	Ref		
Feed source	Exotic	0.851	0.572–1.267	0.427
	Local	Ref		
Clean water	None	0.700	0.371–1.322	0.272
	Disinfection	1.363	0.834–2.227	0.217
	Filter	Ref		

### Occurrence risk of NDV

We use a random forest algorithm to establish an occurrence risk model that includes 8 risk factors. There were 1,065 records in the model and 130 of them were positive, the mtry and ntree values were 6 and 300, respectively. The model adjusted mtry and ntree values to maximize the accuracy of the model. The accuracy of the model was 90.81%, indicating that the model can effectively predict the risk of NDV infection. The model predicted several farming situations with a probability of infection exceeding 80% (Table 5).

**Table 5:** Risk prediction of NDV occurrence under different aquaculture conditions.

Farm type	Housing	Feed type	Feed source	Water source	Clean water	Waste management	Dead poultry disposal	Occurrence risk (%)
1	2	2	1	1	2	1	2	93.28
1	1	2	2	1	3	2	1	89.64
1	1	2	2	1	3	2	1	89.43
1	1	2	2	1	3	2	1	88.39
1	1	2	2	1	3	2	1	88.23
1	1	2	1	1	2	1	2	87.82
1	1	2	1	1	2	1	2	87.05
1	1	2	1	1	2	1	2	86.91
1	1	2	2	1	2	1	2	86.79
1	1	2	2	1	2	1	2	86.76
1	1	2	2	1	2	1	2	86.10
1	1	2	2	1	3	2	1	86.00

Farm type: 1 = Layer farm; Housing: 1 = Indoor housing with some free-range access, 2 = Intensive; Feed type: 2 = Formula feed; Feed source: 1 = Exotic, 2 = Local; Water source: 1 = Wells; Clean water: 2 = Disinfection, 3 = Filter; Waste management: 1 = Sale, 2 = Ferment; Dead poultry disposal: 1 = Harmless treatment, 2 = Bury.

### Virus isolation and sequence analysis

**Figure 6:**
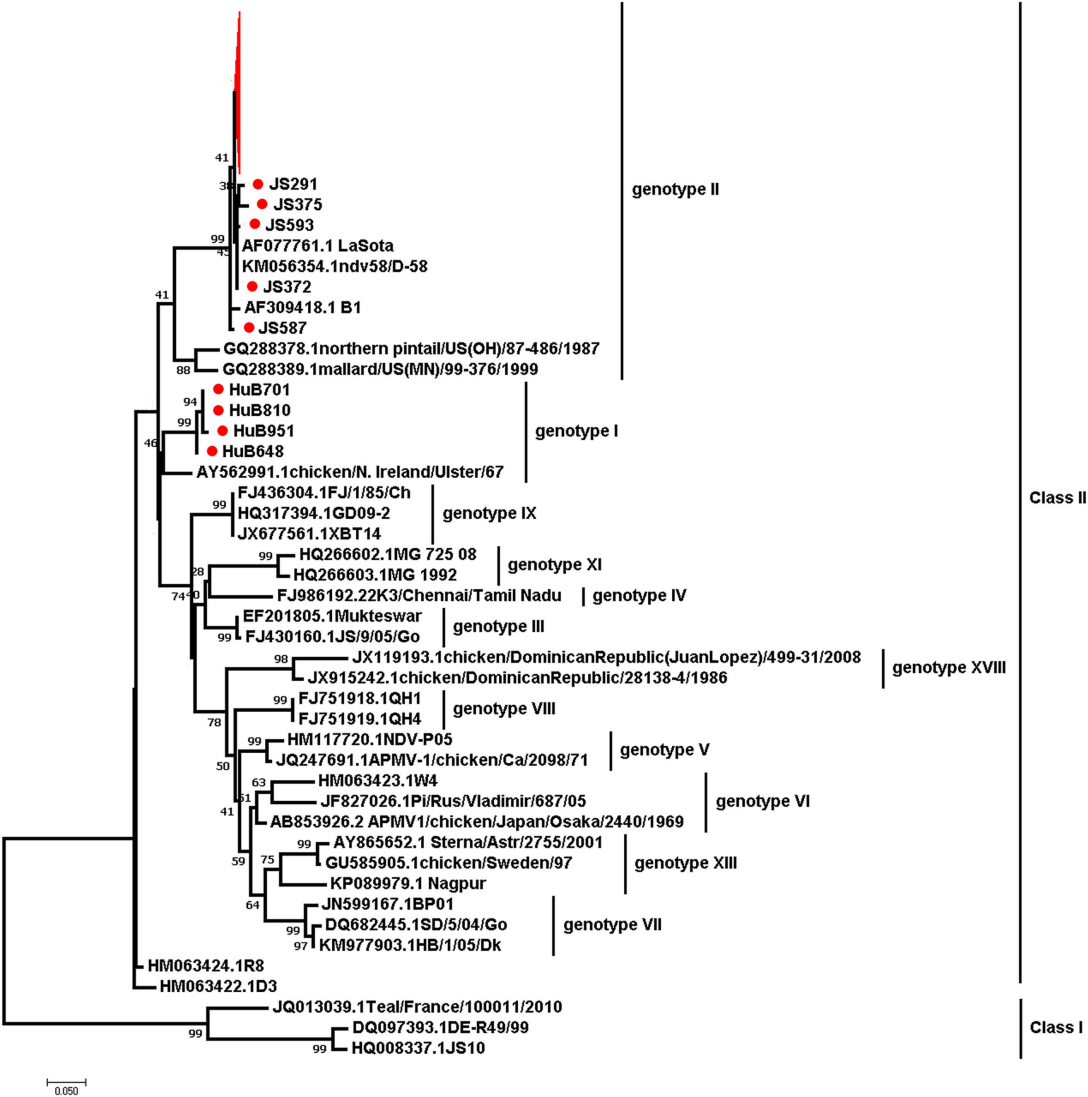
The maximum-likelihood phylogenetic tree based on the partial F gene sequences of NDVs isolated in this study and 34 reference strains. Red branches and dots mark the viruses isolated in this study.

To determine the genotypes of the circulating NDVs in chicken populations, we isolated NDV from 428 RT-PCR-positive samples in SPF eggs. We obtained 104 NDVs and sequenced their F genes. We submitted the sequences to the GenBank (Accession No: PP238275-PP238378). A phylogenetic analysis, based on the F gene nucleotide sequences of these viruses and 34 reference strains from the GenBank database, revealed that all the newly isolated viruses belonged to Class II but were divided into two genotypes. Four isolates from Hubei Province were assigned to genotype I, while the remaining 100 isolates from Shandong Province (9) and Jiangsu Province (91) were assigned to genotype II, which also included the low pathogenic NDV strain LaSota (Figure 6). This suggests that the dominant circulating NDVs are of low pathogenicity to chickens.

## Discussion

Vaccines are widely used on chicken farms in China to prevent NDV infection. However, ND remains a major issue causing significant losses in the poultry industry. Therefore, close monitoring of NDV’s circulation is crucial for adapting disease control policies. Our four-year investigation showed that NDV is relatively well controlled in China. The overall low bird- and farm-level prevalence were about 1 and 10% respectively, which are lower than in countries like Egypt and Vietnam, and similar to India and Africa (21–23). We observed a decrease in bird-level prevalence, suggesting improved farm management. However, there were significant differences at the provincial level, with farm-level prevalences ranging from 0% to approximately 90%. This suggests that NDV circulation remains severe in certain regions. Positive farms were mainly concentrated in Hainan, Heilongjiang, Hubei, and Jiangsu provinces, this may be closely related to the aquaculture situation in these provinces. These four provinces have a large number of livestock and poultry products (data sources from https://www.stats.gov.cn/sj/ndsj/) and the breeding situation was complex. Consequently, NDV control should primarily focus on reducing farm-level infections in these extensively affected regions.

Although the overall prevalence of NDV at bird- and farm-level remained stable, it varied at the provincial level. A logistic regression analysis was conducted to identify potential risk factors associated with NDV infections on farms. The analysis revealed that the prevalence of NDV in layer and broiler farms was significantly higher (*p* < 0.05) than in breeding farms, which is likely the result of good biosecurity practices in breeding farms. Farms where chickens were raised indoors but had some free-range outdoor access were found to have 1.788 times higher odds of NDV infection (*p* = 0.02) than intensive farms. This suggests that outdoor access may increase the chance of effective contact between poultry and carriers of the pathogen, such as infected poultry and wild birds, or contaminated personal and other materials (22). Farms that used mixed feed observed a 2.973 times higher rate of NDV infection (*p* < 0.01) than those using formula feed. Compared to mixed feed, commercial complete formula feeds contain complete nutritional components, which may be more effective in improving poultry’s physical fitness, thereby increasing their resistance to viral infections. To reduce the prevalence of NDV infection in provinces with high farm-level prevalence, these identified risk factors could be targeted for control policy improvement.

The random forest algorithm has been widely used in the field of biology and it has been successfully applied to predict the risk of various diseases occurring (19, 24, 25). The accuracy of the model prediction in this study was 90.81%, indicating that the model can effectively predict the occurrence of NDV infection in different farms. As monitoring continues, the supplemented dataset can continuously optimize model parameters and improve model accuracy. Model prediction results will provide support for disease warning and reference for disease prevention and control.

In this study, we learned about the situation of the farm through the questionnaire, but did not consider the environmental factor data, which may lead us to ignore some risk factors related to the spread of NDV. If we obtained the data of environmental factors through RS and use the GIS spatial analysis tool, it is possible to grasp the spatial and temporal distribution characteristics of diseases and more accurately explain the relationship between NDV and risk factors (26). It is also possible to optimize the occurrence risk model to provide more accurate early warning information.

Based on the F gene’s phylogeny, NDVs have been divided into at least 20 subtypes. Previous studies found that all NDV genotypes have been identified in China, with most recently circulating NDVs primarily from genotype VII (11, 27). In contrast, our study revealed geographical differences among NDV genotypes. Of these 104 NDVs isolated in this study, four belonged to genotype I, and the rest were predominantly from genotype II. These were genetically closely related to the low pathogenic strain LaSota, which differs from other studies findings. This discrepancy may be because our samples were primarily from clinically healthy chickens, unlike other studies aimed at isolating NDVs from diseased chickens, which increases the chance of obtaining virulent strains (28, 29). In recent years, virulent NDVs from genotype VI, VII VIII, IX, and XII have been sporadically reported, but there are few reports on the isolation of genotype I viruses in China (15, 16, 22, 30, 31). Phylogenetic analysis of NDV has important guiding significance for understanding virus mutations and vaccine development. Therefore, long-term monitoring is necessary to prevent outbreaks of diseases.

## Conclusion and recommendations

From 2019 to 2022, a prevalence of NDV at farm-level was high. This means that ND continues to be endemic in different provinces of China and pose a threat to the flock. Farms infection with NDV could be attributed to these factors: layer farm, broiler farm, using mixed feed, and indoor housing with some free-range access. These results provide a reference for ND prevention and control. Hence, continuous pathogen surveillance in poultry flocks should be conducted to prevent the spread of low-pathogenicity strains, and preventive measures should be improved based on risk factors.

## Data Availability

The data that support the findings of this study are available on request from the corresponding author (wangjingfei@caas.cn), upon reasonable request.

## Footnotes

^1^ https://github.com/mrunali-pawar/RandomForest-R-CreditRisk
